# Further evidence for the cognitive disruption and self-talk frequency hypothesis

**DOI:** 10.3389/fpsyg.2026.1716835

**Published:** 2026-02-05

**Authors:** Thomas M. Brinthaupt, Michael J. Connelly, Francesca Mallia

**Affiliations:** Department of Psychology, Middle Tennessee State University, Murfreesboro, TN, United States

**Keywords:** self-talk, cognitive disruption, weak sense of self, mindful awareness, self-control, dissociative experiences, antisocial personality, psychopathology

## Abstract

**Objective:**

Past research has shown support for a positive relationship between cognitive disruption and self-talk frequency in response to specific situations. In this paper, we report three studies that examine further the cognitive disruption and self-talk frequency hypothesis among college students. We sought to identify facets of decreased or increased cognitive disruption that should be associated with either less or more frequent self-talk.

**Methods:**

In Study 1, participants (*N* = 262) completed measures of self-talk, weak sense of self, and mindful awareness. In Study 2, frequent and infrequent self-talk participants (*N* = 54) completed measures of dissociative tendencies and self-control. In Study 3, participants (*N* = 224) completed self-talk, positive and negative automatic thoughts, antisocial personality, and psychopathy measures.

**Results:**

Study 1 results showed that, as expected, individuals with a weaker sense of self and lower levels of mindful awareness reported higher frequencies of self-talk. Study 2 results showed that frequent self-talkers reported significantly more dissociative experiences and higher levels of self-control than infrequent self-talkers. Study 3 results showed that higher antisocial personality and psychopathy scores were significantly related to more frequent negative and self-critical self-talk.

**Conclusion:**

These studies provide strong support for the hypothesis that instances of cognitive disruption are associated with an increased likelihood of self-talk in response to specific situations. We also showed that factors that should be associated with reduced cognitive disruption were associated with less frequent self-talk. We discuss the implications of these findings for future research in personality psychology.

## 1 Introduction

Research on individual differences in self-talk frequency has shown that such differences are strong and pervasive (e.g., Brinthaupt, 2019; Brinthaupt et al., 2015; Oleś et al., 2020). Across a variety of measures of intrapersonal communication, individual variations are common, with some respondents indicating that they rarely or never talk to themselves and other respondents indicating that they talk to themselves very often or “all the time” (e.g., Alderson-Day et al., 2018; Heavey and Hurlburt, 2008).

To organize research on individual differences in self-talk, Brinthaupt (2019) proposed two hypotheses. The social isolation hypothesis predicts that characteristics or experiences that are socially isolating (e.g., living alone, lacking social skills) will be associated with more frequent self-talk. Cognitive disruption refers to states (situations) or traits (typical individual patterns) that reflect experiences such as confusion, feeling overwhelmed, being surprised, and/or having anomalous, upsetting or disturbing self-related experiences. Because there is not currently a direct measure of cognitive disruption (and such disruption is likely to be dependent on the specific situation under consideration), its presence is inferred.

The cognitive disruption hypothesis predicts that anomalies, upsets, and disturbances of experience will be associated with greater self-talk frequency. The rationale here is that disruptive self-related experiences should encourage self-regulatory efforts (e.g., due to factors such as attentional lapses, intrusive thoughts, or identity instability) to address or manage those experiences. This idea is consistent with proposals that self-talk aids a variety of cognitive processes such as deliberation, attention, control, and memory (e.g., Fernyhough and Borghi, 2023; Geng et al., 2025; Kompa and Mueller, 2024).

Brinthaupt's (2019) review indicated good support for the social isolation hypothesis and stronger support for the cognitive disruption hypothesis. With respect to the latter, example research findings show that scores on variables such as schizotypy (Brinthaupt et al., 2020), public speaking anxiety (Shi et al., 2015), disordered eating (Roy and Gabińska, 2023), and obsessive-compulsive tendencies (Brinthaupt et al., 2009) are associated with more frequent self-talk in response to specific situations.

To date, there are very few studies that directly examine factors that should be associated with less cognitive disruption and thus less frequent self-talk. In addition, several self-related variables have yet to be studied as they relate to self-talk frequency. The present studies aimed to add to the literature on the cognitive disruption/self-talk frequency hypothesis. Each study received IRB approval, was administered through a commercial online program (Qualtrics), gave informed consent to participants prior to their participation, allocated course research credit for participating in the study, and provided a written debriefing at their conclusion. All participants came from a General Psychology research pool at a large public university in the southeastern U.S. and received course credit for their participation. We conducted all analyses using SPSS version 26.

## 2 Study 1: weak sense of self, mindful awareness, and self-talk

One factor that should be related to self-talk frequency is the clarity, stability, or strength of one's sense of self. Identity dysfunction or the inability to determine who one is (Kaufman and Crowell, 2018; Nielsen and Wright, 2025) is a transdiagnostic mental health problem. For example, Light (2017) argued that having an unclear self-concept makes self-regulation and the pursuit of goals more difficult. Xiao et al. (2025) found that individuals with low self-concept clarity were more likely to engage in rumination (persistent negative thinking about the self) compared to those with high self-concept clarity. Those with low self-concept clarity are also likely to report greater experiences of instability and distress in their daily lives (e.g., Schwartz et al., 2017). In addition, there is evidence that lower self-concept clarity in adolescence and emerging adulthood is related to more frequent internal dialogues (e.g., Oleś and Puchalska-Wasyl, 2012).

Racy and Morin (2024) examined how several self-related variables related to self-talk frequency. Although they did not frame their study as a test of the cognitive disruption hypothesis, several of their findings provide support for that hypothesis. For example, they found that self-concept clarity was significantly and negatively associated with self-critical, self-managing, and social-assessing self-talk, as well as mind wandering (having frequent task-unrelated thoughts). These results suggest that having a clear and stable self-concept leads to fewer instances of cognitive disruption (and therefore less frequent self-talk). They also found that mindfulness acceptance (accepting experiences openly and without judgment) was negatively related to the frequency of self-critical, social-assessing, and self-managing self-talk. This finding is also consistent with the cognitive disruption hypothesis, given that mindfulness acceptance in part assesses ways that individuals might quiet their inner voice.

A weak sense of self was operationalized by Flury and Ickes (2007) as reflecting a lack of self-understanding, sudden shifts in self-related experiences, confusing one's thoughts and feelings with those of other people, and feeling the tenuousness of one's existence. Research using their Sense of Self Scale shows that individuals with a weak sense of self report lower scores on identity achievement (Ickes et al., 2012), higher scores on Dark Triad traits (Doerfler et al., 2021), higher scores on social comparison behaviors (Price et al., 2023), and lower scores on self-concept clarity (Suszek et al., 2018). All these circumstances suggest greater cognitive disruption associated with a weak sense of self.

Becoming more mindful may be one way to develop a clearer sense of self. For example, Hanley and Garland (2017) found that trait mindfulness was positively related to self-concept clarity. Brown and Ryan (2003) operationalized mindful attention and awareness as a connection to or recognition of aspects of the present moment (e.g., disagreeing with “I find it difficult to stay focused on what's happening in the present”). In the development and validation of their Mindful Attention Awareness Scale, they showed that a lack of attention and awareness is associated with reduced self-awareness, higher scores on depression, and lower scores on self-esteem.

More recently, researchers have found that a lack of attention and awareness is associated with higher levels of stress and lower levels of subjective wellbeing (e.g., Hepburn et al., 2021). Mindfulness-based interventions have been shown to have small positive benefits for attention and executive control (Yakobi et al., 2021). Grzybowski and Brinthaupt (2022) found that positive self-talk facets (i.e., self-reinforcement and positive automatic thoughts) were associated with higher mindfulness scores, whereas negative self-talk facets (i.e., self-criticism, social-assessment, and negative automatic thoughts) were associated with lower mindfulness scores. Other research shows that inner speech (silent self-talk) can enhance people's awareness of their mind-wandering and introspection of their thoughts (Bastian et al., 2017). Thus, individuals who are more mindful should exercise greater control over potential cognitive disruptions, resulting in less need for self-regulatory self-talk.

Given the findings of past research, we predicted that having a weak sense of self would be associated with experiences of cognitive disruption and would therefore be related to a greater tendency to talk to oneself compared to having a strong sense of self. In addition, we expected that individuals who reported higher levels of mindful awareness and attention, because they should experience fewer instances of cognitive disruption, would show less frequent self-talk in response to specific (especially self-critical and social-assessing) situations.

### 2.1 Method

#### 2.1.1 Participants

Participants were 262 students (179 female, 75 male, eight other or missing) with an average age was 19.62 years (*SD* = 2.69). The majority were first-year students (57%) and white (62%).

#### 2.1.2 Measures

*Self-Talk Scale* (STS; Brinthaupt et al., 2009): the 16-item STS measures the frequency (1 = *never*, 5 = *very often*) of a person's typical silent and aloud self-talk in response to specific situations. The response stem used in this measure is “I talk to myself when…” It includes the facets of social assessment (“I want to analyze something that someone recently said to me”), self-management (“I need to figure out what I should do or say”), self-reinforcement (“something good has happened to me”), and self-criticism (“I'm really upset with myself”). Research shows that the STS has good psychometric properties (see Brinthaupt, 2019). With the current sample, internal consistency values were good for total STS (*a* = 0.91) and each subscale (*a* values ranging from 0.81 to 0.88).

*Sense of Self Scale* (SOSS; Flury and Ickes, 2007): the SOSS is a 12-item measure of a weak sense of self. Sample items include “I wish I were more consistent in my feelings” and “Who am I? is a question that I ask myself a lot.” This measure tapped potential disruptive experiences of feeling confused, overwhelmed, or surprised, as well as having anomalous or disturbing self-related experiences and identity instability. Participants rate the items using a 4-point scale (1 = *very uncharacteristic of me*, 4 = *very characteristic of me*). Higher scores reflect a weaker sense of self, with a possible range of 12–48. Flury and Ickes (2007) report that the SOSS has acceptable psychometric properties. With the current sample, the internal consistency value was good (*a* = 0.80).

*Mindful Attention and Awareness Scale* (MAAS; Brown and Ryan, 2003): the MAAS is a 15-item measure of respondents' lack of attention to and awareness of what occurs in the present moment (e.g., “I find it difficult to stay focused on what's happening in the present”). It excludes items that contain attitudinal or acceptance components. This measure also tapped potential disruptive experiences of feeling confused, overwhelmed, or surprised as well as having attentional lapses. Items are rated using a 6-point frequency scale (1 = *almost always*, 6 = *almost never*). Higher scores indicate greater mindful attention, with a possible range of 15–90. The current sample's internal consistency value was acceptable for this measure (*a* = 0.80).

#### 2.1.3 Procedure

The order of the main measures was counterbalanced across participants, with demographic items appearing at the start of the survey. Participants completed the measures as part of early semester Psychology research pool pretesting sessions. As part of the survey, participants reported their gender, age, year in school, and race/ethnicity.

### 2.2 Results

#### 2.2.1 Descriptive statistics

Scores for the three main measures were as follows—STS: *M* = 56.48, *SD* = 12.58; MAAS: *M* = 52.40, *SD* = 13.36; and SOS: *M* = 23.82, *SD* = 7.72. Skewness and kurtosis values were in the acceptable range for these measures. Comparison of the means to the scale midpoints revealed that STS scores were significantly higher than the midpoint (48), *t*(261) = 10.92, *p* < 0.001, *d* = 0.674. MAAS scores did not differ significantly from the scale midpoint (52.5), *t*(261) = 0.12, *p* = 0.90. Weak SOS scores were significantly lower than the scale midpoint (30), *t*(261) = 12.96, *p* < 0.001, *d* = 0.800. Male-female gender comparisons revealed no significant differences on any of the measures. MAAS and SOS scores were significantly and negatively correlated, *r*(260) = −0.473, *p* < 0.001.

#### 2.2.2 Tests of hypotheses

As Table 1 shows, there was strong support for the cognitive disruption hypothesis with moderate effect sizes. Participants with a weaker sense of self reported significantly more frequent total and subscale self-talk. In addition, participants with higher mindful attention and awareness scores reported significantly less frequent total self-talk and less frequent self-talk on three of the four STS subscales.

**Table 1:** Study 1: self-talk, weak sense of self, and mindful attention and awareness.

**Self-Talk Scale**	**Weak sense of self**	**Mindful attention awareness**
STS total	0.369^***^	−0.292^***^
STS self-criticism	0.381^***^	−0.311^***^
STS self-reinforcement	0.177^**^	−0.082
STS self-management	0.273^***^	−0.249^***^
STS social assessment	0.346^**^	−0.292^***^

N = 262. STS, Self-Talk Scale. ^**^p < 0.01. ^***^p < 0.001.

We calculated a linear regression with SOS and MAAS scores as standardized predictors of STS scores. Checking assumptions revealed that linearity, independence of errors, homoscedasticity, normality of residuals, and multicollinearity were all in the acceptable range. The analysis revealed a significant overall model (*R*^2^ = 0.154, *F*(2, 259) = 23.62, *p* < 0.001). Participants' predicted self-talk scores were equal to 52.42 + 0.484 (weak sense of self) – 0.143 (mindful awareness). The model showed that both SOS scores (β = 0.298, *p* < 0.001) and mindful awareness scores (β = −0.152, *p* = 0.020) significantly predicted self-talk scores in the expected direction.

### 2.3 Discussion

In this study, we showed that having a strong sense of self and being mindfully aware and attentive were associated with less frequent self-talk in response to specific situations. In other words, traits such as these are likely to generate fewer instances of cognitive disruption or compensatory cognitive activity that self-talk might help to address.

The sense of self results are consistent with research showing that self-talk clarity is negatively associated with self-talk frequency (Racy and Morin, 2024). However, research also shows that higher authenticity scores (i.e., a clear understanding of one's core self) are associated with more frequent identity internal dialogues (Puchalska-Wasyl, 2022). That result suggests that identity-related self-talk can be useful for clarifying one's sense of self. In other words, the use of self-talk in the work of clarifying one's identity may be more complicated than simply less or more self-talk. Examining individuals with a very weak sense of self would be an interesting extension of the present study. It is possible that a very weak sense of self or a state of identity diffusion might be associated with less frequent self-talk, if such individuals are not motivated to clarify their self or identity.

Future research might examine naturally occurring situations that threaten a person's sense of self or identity to see if there are increases in overall or specific kinds of self-talk. For example, Slepian and Jacoby-Senghor (2021) found that everyday identity threats frequently involve reduced feelings of belonging and increased feelings of exclusion. The feelings of inauthenticity and negative affect associated with such threats are likely to be cognitively disruptive and should be related to more frequent self-talk in response to those threats.

It is also possible that identity opportunities, such as for self-enhancement or moving toward desired states (Bataille and Vough, 2022), will be related to more frequent self-talk. Such situations likely involve efforts to alter or clarify one's self-concept. Examining how instances of identity threat and opportunity relate to self-talk frequency would provide potential support for the cognitive disruption/self-talk frequency hypothesis beyond that found with the more common trait studies.

The mindful attention and awareness results suggest that individuals who are more attentive to and aware of the present moment have less of need for self-talk in response to specific situations. However, different facets of mindfulness appear to have different relationships with self-talk frequency. An alternative measure of mindfulness, the Philadelphia Mindfulness Scale (PHLMS; Cardaciotto et al., 2008), differentiates between mindful awareness (continually monitoring present experience) and acceptance (a non-judgmental attitude of openness toward experiences). Morgan et al. (2020) found that higher acceptance subscale scores tended to be related to lower scores on symptom measures, whereas awareness scores were unrelated to symptom scores.

Assuming that higher symptom scores are associated with greater cognitive disruption, lower mindful acceptance scores should be related to more frequent self-talk. In support of this speculation, Racy and Morin (2024) found negative correlations between PHLMS acceptance and self-talk/inner speech, suggesting that acceptance involves quieting the inner voice. In contrast to the present results, Racy and Morin also found small positive correlations between subscales on the STS and PHLMS awareness scores. Further research assessing how the facets of mindfulness relate to self-talk frequency as well as how differences between meditators and non-meditators (e.g., Somaraju et al., 2023) relate to self-talk frequency would be valuable.

## 3 Study 2: self-talk, dissociative tendencies, and self-control

Another personality variable that should provide a good test of the cognitive disruption/self-talk frequency hypothesis is dissociative experiences. Such experiences are characterized by disconnections or disruptions in people's behavior, awareness, perception, and identity (Lyssenko et al., 2018), often due to traumatic experiences and unconscious processes (e.g., Frewen and Lanius, 2006). Individuals with dissociative experiences report difficulties in integrating conscious states (Basso et al., 2024). Those who report more dissociative experiences are more likely to have dissociative disorders (Lynn et al., 2014; Lyssenko et al., 2018).

Ford (2013) argues that dissociative experiences represent “fundamental alterations in self-regulation” (p. 237) that include “disorganization of the self's ability to maintain a unified repertoire of perceptions, emotions, and cognitions” (p. 239). In support of the cognitive disruption/self-talk frequency hypothesis, Alderson-Day et al. (2018) found that dissociation scores were weakly but positively related to self-talk that showed evaluative (self-critical) elements, the presence of other people, and dialogicality (back-and-forth conversation). Research and theory therefore suggests that individuals with higher levels of dissociative experiences should report more frequent self-talk to deal with the potentially disruptive aspects of those experiences.

Self-control broadly refers to the regulation of thoughts, emotions, and behaviors and includes the ability to delay gratification and resolve competing goals (Duckworth et al., 2019). Research shows that higher levels of self-control are associated with better school and work performance, interpersonal functioning, and wellbeing (e.g., De Ridder et al., 2012; Duckworth et al., 2019). As such, self-control provides another good test of the cognitive disruption/self-talk frequency hypothesis.

Competing hypotheses are possible with self-control and its relationship to self-talk frequency. On the one hand, individuals with high self-control may experience fewer instances of cognitive disruption (and report less frequent self-talk) compared to individuals with low self-control. That is, those with lower self-control levels may need to utilize self-talk more often than those with higher self-control levels, if their low self-control gets them into problematic personal or social situations and they need to resolve those. Potentially supporting this logic, Fedele and Converse (2024) found that lower self-control scores were associated with more frequent and more severe workplace interruptions and intrusions.

On the other hand, people who are higher in self-control may report more frequent self-talk in support of their self-regulatory efforts, regardless of whether they experience episodes of cognitive disruption. Research shows that blocking inner speech hinders self-control and performance, potentially making it more difficult to restrain impulses (e.g., Nedergaard J. S. et al., 2023; Tullett and Inzlicht, 2010). Researchers (e.g., Galanis et al., 2023; Hatzigeorgiadis and Galanis, 2017; Nedergaard J. et al., 2023) argue that one of the important functions of self-talk is to regulate attention and counter the effects of potential distractions. Thus, the active use of self-talk may be an important self-regulatory tool to help people's self-control efforts.

In summary, in this study we expected that more frequent dissociative experiences would be associated with more frequent self-talk, assuming that such experiences increase the likelihood of cognitive disruption. We also expected that self-control would be related to self-talk frequency, with either a negative or positive relationship possible.

### 3.1 Method

#### 3.1.1 Participants

Participants were General Psychology undergraduates who took part in a departmental pretesting session conducted at the start of the academic term. That session included the Self-Talk Scale as well as a variety of other personality and demographic variables. We used extremes on the STS to provide a stronger test of our hypotheses (see Brinthaupt et al., 2015). Based on the upper and lower quartiles of total STS scores, we recruited 54 participants (34 female, 20 male) to attend another session approximately 1 month later. The final sample had 35 frequent self-talkers (20 female; *M*_*STS*_ = 65.94, *SD* = 7.11) and 19 infrequent self-talkers (14 female; *M*_*STS*_ = 37.32, *SD* = 5.77). The majority were first-year students (48%) and white (63%). They received course credit for both research sessions.

#### 3.1.2 Measures

*Dissociative Experience Scale II* (DES-II; Carlson and Putnam, 1993): the DES-II is a 28-item measure of dissociative tendencies and experiences. The items assess how often respondents have experiences in their daily life, while they are not under influence of alcohol or drugs. Respondents rate each item using an 11-point frequency scale (0% = *never*; 100% = *always*). Most items begin with the phrase “Some people have the experience of…” The DES-II includes three subscales: amnesia (memory and awareness gaps; e.g., “finding new things among their belongings that they do not remember buying”), depersonalization/derealization (feeling of detachment or unreality; e.g., “feeling that their body does not seem to belong to them”), and absorption/imaginative involvement (being highly engaged in an activity with reduced awareness of one's surroundings; e.g., “being in a familiar place but finding it strange and unfamiliar”). Subscales are calculated by reducing each score by one decimal, then averaging the items for that subscale, with possible scores ranging from 0 to 10. The DES-II is a frequently used measure, with good psychometric properties (Arzoumanian et al., 2023; Carlson and Putnam, 1993). This measure tapped potential disruptive experiences of feeling confused, overwhelmed, or surprised, as well as having attentional lapses and anomalous or disturbing self-related experiences. For the current sample, internal consistency values for the total and subscale scores were acceptable (see Table 2).

**Table 2:** Study 2: dissociative experience and self-control scores for frequent and infrequent self-talkers.

**Measure**	**Frequent self-talkers (*****n*** = **35)**		**Infrequent self-talkers (*****n*** = **19)**		***t***	***p***	***d***	**α**
	***M***	***SD***	***M***	***SD***				
Dissociative Experiences Scale II (total)	3.21	1.50	2.34	1.59	1.999	0.025	0.570	0.935
Amnesia	1.80	1.54	1.49	1.71	0.689	0.247	0.196	0.867
Depersonalization/derealization	2.00	1.77	0.98	1.65	2.077	0.021	0.592	0.871
Absorption/imaginative involvement	5.10	2.04	3.73	1.85	2.431	0.009	0.693	0.844
Self-Control Scale	114.57	11.48	106.37	12.12	2.460	0.009	0.701	0.653

N = 54. Self-talk groups based on upper and lower Self-Talk Scale quartiles; Dissociative Experiences Scale possible range is 0–10; Self-Control Scale possible range is 36–180.

*Self-Control Scale* (SCS): the SCS (Tangney et al., 2004) is a 36-item unidimensional measure of the extent to which people regulate themselves to achieve long-term goals. It focuses on people's ability to refrain from undesired behaviors and control inner responses. This measure tapped experiences that are likely to reduce feeling confused, overwhelmed, or surprised, as well as limit the likelihood of having attentional lapses and anomalous or disturbing self-related experiences. Items are rated on a 5-point scale (1 = *not at all*, 5 = *very much*) based on how much each statement reflects respondents' typical behaviors or perceptions. Sample items include “I am good at resisting temptation” and “I wish I had more self-discipline” (reversed). Possible scores ranged from 36 to 180, with higher scores denoting greater levels of self-control. With the current sample, coefficient alpha was acceptable for the measure (see Table 2).

#### 3.1.3 Procedure

In the follow-up session, we invited students to participate in “a study that examines everyday experiences and how people ‘regulate' or adjust themselves as they go about their daily activities.” They completed the measures on the computer in groups of 2–4. No mention was made of their previous self-talk scores or why they were selected to participate in this session. Two researchers in this second phase were blind to the participant group (frequent or infrequent self-talk) as they completed the survey. Participants received the DES-II and the SCS in random order.

### 3.2 Results

#### 3.2.1 Descriptive statistics

Table 2 provides the descriptive statistics for the frequent and infrequent self-talkers for the DES-II and SCS scores. Skewness and kurtosis values were in the acceptable range for these measures. Participants reported DES-II total scores (*M* = 2.91, *SD* = 1.58) that were significantly below the midpoint (5.50) of the scale, *t*(53) = 12.10, *p* < 0.001, *d* = 1.58. Among the subscales, scores were low for amnesia (*M* = 1.69, *SD* = 1.65) and depersonalization (*M* = 1.64, *SD* = 1.78) but higher for absorption (*M* = 4.61, *SD* = 2.06). Data for the SCS indicated that participants scored significantly above (*M* = 111.69, *SD* = 12.25) the midpoint (90) of the measure, *t*(53) = 13.01, *p* < 0.001, *d* = 1.77. Male-female gender comparisons on the main measures revealed only one significant difference, with male participants (*M* = 5.36, *SD* = 2.24) reporting higher DES-absorption scores than female participants (*M* = 4.18, *SD* = 1.85), *t*(53) = 2.10, *p* = 0.04, *d* = 0.591.

#### 3.2.2 Tests of hypotheses

One-tailed *t*-tests revealed that the frequent self-talkers reported significantly higher total, depersonalization, and absorption dissociation scores than infrequent self-talkers. Results also showed that frequent self-talkers scored significantly higher in self-control compared to infrequent self-talkers. In other words, individuals with higher dissociative and self-control tendencies reported more frequent self-talk in response to specific situations, showing medium effect sizes.

Analysis of the correlations among the main measures revealed that self-control scores were significantly and positively related to total DES (*r*(52) = 0.320, *p* = 0.018) and DES-absorption (*r*(52) = 0.357, *p* = 0.008) scores. That is, individuals with more frequent dissociative experiences reported higher levels of self-control. STS scores were significantly related to self-control scores (*r*(52) = 0.374, *p* = 0.005). Total STS scores were positively correlated with total dissociation scores (*r*(52) = 0.241, *p* = 0.079) as well as significantly with DES-absorption (*r*(52) = 0.300, *p* = 0.026). In addition, STS self-reinforcement scores were significantly correlated with total DES (*r*(52) = 0.296, *p* = 0.030), DES-depersonalization (*r*(52) = 0.272, *p* = 0.047), and DES-absorption (*r*(52) = 0.311, *p* = 0.022). STS self-managing scores were significantly associated with DES-absorption (*r*(52) = 0.298, *p* = 0.029).

We calculated a linear regression with total DES-II and SCS scores as standardized predictors of total STS scores. A check of the assumptions revealed that linearity, independence of errors, homoscedasticity, normality of residuals, and multicollinearity were all in the acceptable range. The analysis revealed a significant overall model (*R*^2^ = 0.156, *F*(2, 51) = 4.72, *p* = 0.013). Participants' predicted self-talk scores were equal to 5.939 + 0.413 (self-control) + 1.316 (dissociative experiences). The model showed that self-control scores significantly predicted total self-talk scores (β = 0.330, *p* = 0.018), whereas dissociative experience scores did not (β = 0.136, *p* = 0.323). Testing a model with the DES-II subscales rather than the total score showed a similar effect, with none of the subscales significantly predicting self-talk scores.

### 3.3 Discussion

In Study 2, we compared the characteristics of individuals who reported frequent and infrequent self-talk. With respect to dissociative experiences, the results provided good support for the cognitive disruption/self-talk hypothesis. Individuals who talk more often to themselves in response to specific situations reported more frequent dissociative experiences. In addition, higher self-control scores were associated with more frequent self-talk, supporting the proposition that self-talk is an important self-regulatory tool.

The dissociation results suggest that different facets may be differentially related to self-talk frequency and raise questions about causality. We found that the amnesia facet of the DES-II was weakly related to self-talk frequency. This facet refers to memory disruptions (e.g., failure to recall important events or personal information). Theoretically, memory disruptions should lead to greater instances of cognitive disruption (e.g., trying to recall what happened, resolving evidence that contradicts one's memory, etc.). However, the nature of dissociative amnesia may induce a focus or emphasis on the past rather than the present, which could be less cognitively disruptive or be less likely to induce a need for self-regulation.

The depersonalization/derealization facet of the DES-II reflects self-related and external environment disturbances that include feelings of detachment or unreality. Depersonalization also has been conceptualized as a defensive and protective response to threat or stress (e.g., Sierra et al., 2002). As such, increased self-talk frequency could reflect efforts to regulate disturbances or inhibit responses to stressors. Alternatively, more frequent self-talk might make depersonalization experiences more likely.

The absorption (or imaginative involvement) facet of the DES-II includes being highly engaged in an activity and experiencing a familiar place as strange or unfamiliar. These experiences should foster increased self-regulatory efforts to maintain that involvement or understand its effects. Alternatively, greater self-talk frequency could make absorption experiences more likely. It is also possible that absorption might reflect a flow state rather than cognitive disruption. For example, Taylor et al. (2018) found that flow experiences were positively related to motivational and positive self-talk frequency among competitive athletes. Future research could clarify causal questions for each of the DES-II facets and explore in greater depth how those facets relate to self-talk frequency and content.

The fact that more frequent dissociative experiences were associated with higher levels of self-control is consistent with the view that self-talk is a tool for self-control and self-regulation (e.g., Carver and Scheier, 1998; Mischel et al., 1996). Given that higher self-control is associated with more frequent self-talk, there are implications for training self-control (Friese et al., 2017) with self-talk interventions, such as those used in sport psychology (e.g., Hatzigeorgiadis et al., 2011; Latinjak et al., 2023).

The positive relationship between dissociative experience and self-control scores indicates that dissociative experiences may be associated with increased self-control efforts through the use of self-talk. Furthermore, the regression analysis showed that self-control scores significantly predicted self-talk scores, whereas dissociative experiences did not. This suggests that the dissociative experiences/self-talk relationship is primarily driven by self-control elements. Basso et al. (2024) found that dissociation is related to a variety of impairments that suggest reduced self-control.

Our sample consisted mainly of individuals who did not have very high scores on dissociative experiences. We also did not assess whether any of our participants had been diagnosed with dissociative identity disorder. The results suggest that individuals with such a disorder should fall into the upper end of self-talk frequency, to the extent that they use self-talk to manage their episodes of cognitive disruption.

Although there are advantages to using an extreme-groups design, there are also some potential limitations. The small sample size of infrequent self-talkers may have been underpowered for some analyses. In addition, because we removed the middle range of STS scorers, we were unable to examine any possible nonlinear relationships between self-talk and dissociative experiences scores. Using extreme groups may limit the reliability of STS scores, since those scores may be more subject to regression to the mean effects compared to middle range STS scores (Preacher et al., 2005). Finally, the internal consistency value for the SCS was weaker than the other measures, so those results must be interpreted with caution.

## 4 Study 3: self-talk frequency and anti-social personality disorder and psychopathy characteristics

Whereas, there is much research on dysfunctional and negative self-talk content among various clinical conditions (e.g., Scott et al., 2014; Treadwell and Kendall, 1996), there is very little research on how self-talk frequency is related to such trait tendencies among nonclinical samples. Examining this relationship provides a valuable test of the cognitive disruption/self-talk frequency hypothesis with individuals who should experience higher levels of cognitive disruption compared to those with lower levels of such traits. In line with this speculation, Petrolini et al. (2020) reviewed evidence that intrusive and uncontrollable inner speech (which should be cognitively disruptive) inhibits executive function for individuals with schizophrenia experiencing auditory hallucinations. In this study, we chose measures of anti-social personality and psychopathy under the assumption that these characteristics provide a range of potentially disruptive cognitive elements.

Those with antisocial personality disorder (ASPD) display a range of criminal and unhealthy or inappropriate interpersonal behaviors and have difficulty maintaining goal directed behavior (e.g., Black, 2024; McGonigal and Dixon-Gordon, 2020). People with ASPD struggle with empathy and awareness that others' actions are intentional (Bateman and Fonagy, 2013; Marzilli et al., 2021). Bateman et al. (2013) note that individuals with ASPD spend significant time attempting to cognitively understand their own and other peoples' mental states.

Antisocial personality characteristics are marked by impulsive, irrational behaviors, decrements in verbal, analytic brain processes, and disjunctions between thoughts and action (Snow and Thurber, 1997; Swann et al., 2009). These characteristics also show significant overlap with other kinds of psychopathy (Anderson and Kelley, 2022; Glenn et al., 2013). Validating a measure of cognitive distortions (measuring common cognitive errors), Morrison et al. (2015) found that scores on that measure correlated significantly with measures of automatic thoughts, depression, worry, and social interaction anxiety. All of these findings suggest that higher levels of anti-social personality characteristics should be associated with greater cognitive disruption.

Psychopathy is a personality trait that is similar to antisocial personality disorder. It includes deficits in the interpersonal and affective domains, antisocial tendencies, and impulsivity and sensation-seeking (Hare, 2006). Individuals with psychopathic traits experience difficulties in processing reward and punishment contingencies and other kinds of decision-making (Atanassova et al., 2025).

De Brito et al. (2021) note that psychopathy is characterized by neurocognitive disruptions or disturbances in emotional responsiveness (i.e., deficits in empathy and perceptions of fear), attention (i.e., compromised selective attention), and reinforcement-based decision-making (i.e., being impulsive and making poor decisions). Those with high psychopathy scores are also likely to be confronted with their own weaknesses and experience dissatisfaction with their deviant behavior (Martens, 2014). The experiences of those with psychopathy characteristics provide an additional good test of the cognitive disruption/self-talk frequency hypothesis.

Given past research showing that negative and dysfunctional self-talk relates to a variety of clinical and other conditions (e.g., Grzybowski and Brinthaupt, 2022; Treadwell and Kendall, 1996), we expected that self-talk content (i.e., positive and negative self-talk) would show stronger relationships with clinical trait tendencies compared to self-talk frequency. In particular, we expected strong positive correlations between antisocial personality and psychopathy scores and negative automatic thoughts, and strong negative correlations of antisocial personality and psychopathy with positive automatic thoughts.

We also expected that various facets of clinical trait tendencies would be related to more frequent overall self-talk. Because of the manipulative nature of those with antisocial personality and psychopathy characteristics, we predicted that those scoring higher on these characteristics would report higher levels of social-assessing self-talk. In addition, given the potential negative experiences and disruptions experienced by those with antisocial personality and psychopathy tendencies, we expected that higher scores on these characteristics would be associated with more frequent self-critical and less frequent self-reinforcing self-talk.

### 4.1 Method

#### 4.1.1 Participants

The sample included 224 undergraduate students (158 female, 59 male, seven other) with an average age of 19.86 years (*SD* = 3.92). Most participants were White (59%).

#### 4.1.2 Measures and procedure

*Self-Talk Scale* (STS; Brinthaupt et al., 2009): see Study 1 for a description of the STS. With the present sample, internal consistency values were good for total STS and each subscale (see Table 3).

**Table 3:** Study 3: descriptive statistics for the major measures.

**Major measures**	***M***	***SD***	***a***
**Self-Talk Scale**			
Total	59.04	11.39	0.89
Self-criticism	14.67	3.96	0.85
Self-reinforcement	12.99	3.78	0.82
Self-management	16.04	3.28	0.76
Social assessment	15.34	3.97	0.84
**Automatic Thoughts Questionnaire-Revised**			
Positive	29.54	7.78	0.88
Negative	68.32	27.83	0.97
**Antisocial Personality Questionnaire**			
Self-control	0.932	0.035	0.55
Low self-esteem	0.549	0.253	0.85
Avoidance	0.666	0.212	0.68
Paranoid	0.473	0.194	0.75
Aggression	0.658	0.145	0.49
Deviance	0.548	0.160	0.70
Extraversion	0.655	0.146	0.65
Resentment	0.533	0.204	0.76
**Expanded Levenson Self-Report Psychopathy Scale**			
Egocentric	1.82	0.474	0.78
Callous	1.76	0.482	0.80
Antisocial	1.99	0.472	0.80

N = 224. STS subscale possible scores ranged from 4 to 20; positive ATQ-R possible scores ranged 10–50; negative ATQ-R possible scores ranged from 30 to 150; statistics for the APQ reflect averages ranging from 0 to 1; statistics for the E-LSPS subscales reflect averages ranging from 1 to 4.

*Automatic Thoughts Questionnaire-Revised*: the Automatic Thoughts Questionnaire-Revised (ATQ-R; Kendall et al., 1989) is a 40-item scale measuring positive (10 items) and negative (30 items) automatic thoughts that have occurred over the past week. It reflects self-talk content rather than self-talk frequency. Example negative items include “I am a failure” and “I can't get things together.” Example positive items include “I feel very happy” and “This is super!” Respondents rate the items using a 5-point frequency scale (1 = *not at all*, 5 = *all the time*). Items are summed to create the positive and negative subscales. Possible scores range between 10 and 50 for the positive items and between 30 and 150 for the negative items. Research (e.g., Burgess and Haaga, 1994; Kendall et al., 1989) provides evidence for the measure's reliability and validity. With the current sample, internal consistency values were good (see Table 3).

*Antisocial Personality Questionnaire* (APQ): the APQ (Blackburn and Fawcett, 1999) is a 125-item measure with eight subscales consisting of yes/no questions, originally developed for assessing deviance in offender populations but also frequently used with college students. The *self-control* subscale consists of 20 items referring to the denial of inappropriate behaviors, feelings, and interpersonal reactions (e.g., “Do you sometimes feel like swearing?”), with higher scores denoting overcontrol or rigid conformity. *Self-esteem* (18 items) refers to feelings of dysphoria, helplessness, and self-denigration (e.g., “Do you sometimes feel extremely useless?”), with higher scores reflecting negative or low self-esteem. *Avoidance* (16 items) refers to evading and having difficulties in social interactions (e.g., “Do you often wish you weren't so shy?”), with higher scores denoting social withdrawal and lack of social skills. *Paranoid suspicion* (17 items) represents an increased tendency to show suspicion and vigilance of others and beliefs that others have ill intent toward oneself (e.g., “Do you feel that you're being talked about?”).

The *resentment* APQ subscale (19 items) refers to mistrust of others (e.g., “Do you often find yourself disagreeing with people?”), with higher scores reflecting the tendency to blame others. *Aggression* (20 items) reflects the propensity for getting angry, impatient, and physically aggressive (e.g., “When you lose your temper are you capable of slapping someone?”), with higher scores reflecting a lack of restraint. *Deviance* (20 items) refers to having an inclination toward or history of rule-breaking, rebelliousness, delinquency, and conflicts in close relationships (e.g., “Have you ever been in trouble with the law?”). *Extraversion* (20 items) represents general outgoingness, sociability, and enjoyment of daily activities (e.g., “Is your daily life full of things that keep you interested?”), with higher scores reflecting positive affect. The APQ tapped a wide range of potential disruptive experiences of feeling confused, overwhelmed, or surprised, as well as having identity instability and anomalous or disturbing self-related experiences. With the current sample, most of the internal consistency values for the APQ subscales were acceptable (see Table 3). Because of differences in the number of subscale items, the reported results reflect average scores for each subscale.

*Expanded Self-Report Psychopathy Scale*: the expanded version of the three-factor Levenson Self-Report Psychopathy Scale (E-LSRP; Christian and Sellbom, 2016) is a 36-item measure of dimensions of antisociality (13 items; e.g., “I usually can't keep out of trouble for too long”), callousness (12 items; e.g., “I tend not to think about other people's feelings”), and egocentricity (11 items; e.g., “Looking out for myself is my top priority”). We included this measure to tap potential disruptive experiences of feeling confused and having attentional lapses and anomalous or disturbing self-related experiences. Respondents rate the items using a 4-point Likert scale (1 = *disagree strongly*, 4 = *agree strongly*). There is good evidence for the reliability and validity of the E-LSRP (Christian and Sellbom, 2016; Lagera and Sellbom, 2023; Sellbom et al., 2018). With the current sample, internal consistency values for the three subscales were acceptable (see Table 3). Because of differences in the number of subscale items, the reported results reflect average scores for each subscale.

*Procedure*: participants completed the survey online. The order of presentation of the main measures (STS, ATQ-R, APQ, E-LSRP) was counterbalanced across participants. The demographic items (i.e., age, gender, race/ethnicity) always appeared at the end of the survey. All participants correctly answered a quality check item toward the end of the survey.

### 4.2 Results

Table 3 provides descriptive statistics for the major measures. Skewness and kurtosis values were in the acceptable range for all the measures. Participants tended to report more frequent self-managing and less frequent self-reinforcing self-talk compared to self-critical and social-assessing self-talk. The positive automatic thoughts mean was close to the scale midpoint (30). However, the negative automatic thoughts mean was below the scale midpoint (90). The APQ subscales with means above the scale midpoint included self-control, avoidance, aggression, and extraversion. The remaining subscales showed moderate levels. The E-LSRP subscales indicated that the sample did not show particularly high levels of psychopathy (all means below the scale midpoint of 2.50).

Table 4 provides the correlations among the main measures, with reported significance levels subject to a Bonferroni correction (11 tests for each column). The first two columns of the table provide correlations of automatic thoughts (ATQ-R) scores with antisocial personality and psychopathy scores. As the table shows, negative and positive self-talk content was strongly related to those scores in the expected directions. Negative automatic thoughts were positively related to most of the antisocial personality subscales (except for self-control and extraversion) as well as the antisocial psychopathy subscale, with moderate to large effect sizes. Positive automatic thoughts were negatively related to most of the subscales.

**Table 4:** Correlations of automatic thoughts and self-talk scale scores with major measures.

**Major measures**	**Automatic Thoughts**		**Self-Talk Scale**				
	**Negative**	**Positive**	**Self-criticism**	**Self-reinforcement**	**Self-management**	**Social-assessment**	**Total**
**Antisocial Personality Questionnaire**							
Self-control	0.17	−0.19	−0.07	−0.03	−0.11	−0.08	−0.09
Low self-esteem	0.73^**^	−0.55^**^	0.36^**^	−0.15	0.11	0.20^*^	0.18
Avoidance	0.35^**^	−0.40^**^	0.16	−0.10	0.08	0.12	0.09
Paranoid	0.50^**^	−0.20^*^	0.20^*^	0.02	0.14	0.15	0.17
Aggression	0.24^**^	−0.13	0.06	0.01	0.06	0.11	0.08
Deviance	0.51^**^	−0.24^**^	0.24^**^	−0.03	0.04	0.12	0.13
Extraversion	−0.29^**^	0.50^**^	−0.17	0.19	0.02	−0.09	−0.02
Resentment	0.37^**^	−0.06	0.07	−0.02	0.02	0.10	0.06
**Expanded Levenson Self-Report Psychopathy Scale**							
Egocentric	0.19	0.01	−0.02	−0.07	−0.12	−0.03	−0.08
Callous	−0.02	−0.10	−0.18	−0.10	−0.16	−0.08	−0.17
Antisocial	0.48^**^	−0.26^**^	0.22^**^	−0.04	0.16	0.16	0.16

N = 224; Bonferroni correction (11 tests per column): ^*^p < 0.004, ^**^p < 0.001.

The remaining columns of Table 4 provide the correlations of self-talk frequency (STS) scores with antisocial personality and psychopathy scores. As we expected, self-talk frequency was less strongly related to those scores compared to automatic thoughts. However, there were some significant relationships. Specifically, self-critical self-talk showed moderate effect sizes with three antisocial personality subscales as well as the antisocial subscale of the E-LSRP. Social-assessing self-talk frequency was positively related to low self-esteem tendencies. The other self-talk subscales were unrelated to antisocial and psychopathy characteristics. Thus, there was some support for our prediction that self-talk frequency would be related to antisocial and psychopathy characteristics.

Linear regression analyses provided additional information on the observed relationships. Values for linearity, independence of errors, homoscedasticity, normality of residuals, and multicollinearity were all in the acceptable range for the following three tests. A multiple linear regression was conducted to examine whether standardized APQ and E-LSRP scores predicted total Self-Talk Scale (STS) scores. The overall model was statistically significant, *F*(11, 212) = 2.13, *p* = 0.019, and explained 10% of the variance in total STS scores. Two predictors were significant: E-LSRP callous (*B* = −0.31, *SE* = 0.15, β = −0.16, *p* = 0.045) and E-LSRP anti-social (*B* = 0.34, *SE* = 0.16, β = 0.18, *p* = 0.036). The remaining predictors were not statistically significant.

The multiple linear regression model using standardized APQ and E-LSRP scores to predict STS self-criticism was significant, *F*(11, 212) = 4.74, *p* < 0.001, *R*^2^ = 0.20. APQ self-esteem positively predicted self-critical self-talk (β = 0.29, *p* = 0.003), whereas E-LSRP callous traits negatively predicted self-critical self-talk frequency (β = −0.16, *p* = 0.027).

For STS self-management, the regression model was significant, *F*(11, 212) = 2.58, *p* = 0.004, *R*^2^ = 0.12. APQ paranoia (β = 0.27, *p* = 0.016) and E-LSRP antisocial traits (β = 0.26, *p* = 0.003) positively predicted self-management, whereas E-LSRP egocentric traits (β = −0.16, *p* = 0.044) negatively predicted self-management. The remaining predictors were not statistically significant. The models predicting STS self-reinforcement and STS social-assessment were not significant.

### 4.3 Discussion

The main findings from Study 3 indicate that antisocial personality and psychopathy scores are associated with more frequent negative and self-critical self-talk. These results confirm our expectation that these clinical trait tendencies are characterized by highly negative experiences among nonclinical participants. Differences in the magnitude of relationships for self-talk content (positive/negative automatic thoughts) and self-talk frequency (functions served by self-talk) likely reflect the overall negative aspects of antisocial personality and psychopathy traits.

The self-criticism finding is consistent with research showing that this facet of self-talk serves as a transdiagnostic indicator of various kinds of potential cognitive disruption, including self-harm (Zelkowitz and Cole, 2019), eating disorders (Williams and Levinson, 2022), depression (McIntyre et al., 2018), and insecure attachment styles (Rogier et al., 2023).

Callousness was related to less frequent overall and self-critical self-talk. Past research finds that high callousness is associated with a lack of empathy and guilt as well as a tendency toward being cold-hearted, unfeeling, and remorseless (e.g., Anderson and Marcus, 2019; Zych et al., 2019). In other words, such individuals seem to lack caring for others and their diminished self-talk may reflect a lack of care as well as cognitive control impairments (Winters and Sakai, 2023). Reduced self-talk may be due to diminished self-reflection, lower feelings of guilt, or both. Alternatively, less frequent self-talk could increase the chances that such individuals reflect less on themselves and are less prone to feelings of guilt. Future research could clarify the nature and causal direction of the callousness/self-talk negative relationship.

Self-managing self-talk was positively predicted by paranoid and antisocial tendencies. Past research finds that those tendencies are associated with increased cognitive disruption (e.g., Barriga et al., 2008; Saarinen et al., 2021). This result therefore also supports the cognitive disruption/self-talk frequency hypothesis.

The limitations of our sample should be acknowledged. As the descriptive statistics indicated, our college student participants did not score particularly high on the measures of antisocial personality and psychopathy. Our results may reflect normative variability rather than clinical/diagnostic phenomena. Including participants with a diagnosis of ASPD or other clinical conditions would provide a potentially stronger test of the cognitive disruption/self-talk frequency hypothesis.

It is likely that self-talk frequency will vary depending on the nature of the condition. For example, Petrolini et al. (2020) argued that individuals with autism spectrum conditions report reduced use of inner speech compared to neurotypicals, whereas individuals with schizophrenia and auditory hallucinations appear to suffer from uncontrollable and intrusive self-talk. With respect to other clinical issues, research on patients with borderline and avoidant personality disorders (Faggioli et al., 2024; Jørgensen and Bøye, 2022; Weme et al., 2023) suggests a combination of factors that are likely to be cognitively and emotionally disruptive. The cognitive disruption hypothesis predicts that such individuals will report high levels of self-talk frequency as they struggle to clarify their identities.

The Study 3 results suggest that pathological narcissism, not assessed by the measures we used, might be related to increased self-talk frequency. Kaufman et al. (2020) note that neurotic and antagonistic narcissism tendencies were correlated with having a weaker sense of self, along with many other aspects of cognitive disruption. Blay et al. (2024) reported a positive relationship between vulnerable narcissism and emotion dysregulation. Based on findings such as these, it would be interesting to explore the nature and frequency of self-talk among individuals who are high in narcissism. Assuming that such individuals have frequent episodes of cognitive as well as emotional disruption, we would expect that they would report more frequent overall self-talk, particularly more frequent self-critical and social-assessing self-talk. Future research can therefore provide additional clarification for the cognitive disruption/self-talk frequency hypothesis as it relates to different clinical conditions.

## 5 General discussion

The results of these studies provide further support for the cognitive disruption/self-talk frequency hypothesis. The studies highlight the fact that self-talk serves a variety of potential self-regulatory functions, including clarifying one's sense of self, responding to a lack of mindful awareness, coping with dissociative experiences, supporting self-control efforts, and managing negative personality traits. There is now good correlational evidence that states and traits of cognitive stability and equilibrium are related to less frequent self-talk. One potential reason for this relationship is that the need for self-regulation and self-talk as a self-regulatory tool is reduced.

It is conceivable that having frequent self-talk in response to various situations might lead to less clarity for one's sense of self. Similarly, frequent self-talk in response to different situations might enhance tendencies toward showing anti-social personality characteristics. However, if self-talk serves a self-regulatory function (e.g., Kross et al., 2014), then it seems unlikely that situations requiring greater self-regulation would cause anti-social tendencies or a weakened sense of self. Similarly, it seems unlikely that less frequent self-talk in response to specific situations would cause an increase in mindful awareness and attention. The opposite direction of causality appears more likely with each of these variables. Future experimental research is needed to determine whether this is the case.

In none of the studies did we directly measure or manipulate cognitive disruption. With respect to causality, we do not know whether excessive self-talk leads to more frequent cognitive disruptions or more frequent disruptions lead to more frequent self-talk. Clearly additional experimental research (e.g., Kittani and Brinthaupt, 2023) is warranted to determine potential causal paths between cognitive disruption and self-talk frequency.

Because the STS emphasizes self-talk functions rather than self-talk content (e.g., positive or negative, irrational thoughts), additional research is needed to determine whether cognitively disrupting experiences are related to certain kinds of self-talk content. Research reviewed by Brinthaupt (2019) as well as the current results show good support for the hypothesis that cognitive disruption is positively related to self-talk frequency. However, there is very little work assessing self-talk content in response to cognitive disruptions.

The results of Study 3, as well as other studies (e.g., Grzybowski and Brinthaupt, 2022), suggest that cognitive disruptions are associated with increased negative and self-critical self-talk. Without experimental research that manipulates such disruptions, we have no direct evidence that they increase the likelihood that people will talk to themselves as they attempt to navigate those circumstances.

Blackman et al.'s (2024) meta-analysis found that dissociation was moderately and positively related to social isolation schemas (maladaptive cognitive working models related to interpersonal interactions). Our Study 3 findings suggest that psychopathological factors related to social isolation (i.e., APQ low self-esteem and paranoid and E-LSRP antisocial scores) were associated with more frequent self-talk. Thus, there is some potential support for the social isolation hypothesis (Brinthaupt, 2019) in Study 3 as well.

### 5.1 Implications for future research

At present, there is strong evidence that traits that should be associated with greater cognitive disruptions are also related to more frequent self-talk. Future research could include a validated measure of cognitive distortions (e.g., Morrison et al., 2015) or experimentally vary cognitive overload (e.g., Geng et al., 2025) to further test the cognitive disruption/self-talk hypothesis. We expect that high scores on cognitive distortion and experiences of cognitive overload will be positively related to self-talk frequency.

Future research should also distinguish between trait cognitive disruption (as this research has done) and state cognitive disruption (e.g., specific circumstances where a person is confused, stressed, or upset). Instances of state disruption should be associated with stronger self-talk frequency effects, especially given that the STS assesses typical self-talk in response to specific situations.

All the present studies used the STS, a self-report scale that assesses the functions that self-talk serves. Thus, the results do not reflect people's general tendency to talk to themselves irrespective of specific situations (see Brinthaupt et al., 2024). Although self-talk can sometimes be automatic, under-controlled, or outside of a person's awareness (Latinjak et al., 2023), the STS has proven to be a useful measure to assess its self-regulatory aspects. Nonetheless, future research could employ other measures of self-talk related traits (e.g., Alderson-Day et al., 2018; Oleś et al., 2024) to overcome any biases or limitations of the STS in testing the cognitive disruption/self-talk frequency hypothesis.

Some of the measures that we included in this set of studies (e.g., weak sense of self, dissociative experiences, and antisocial tendencies) are likely to include elements of social isolation as well as cognitive disruption (see Brinthaupt, 2019). Rather than operating independently, these two hypotheses might interact or jointly explain variance in self-talk frequency. Exploring traits or situations that are mainly cognitively or emotionally disruptive (or assessing the degree that each kind of disruption is present) would be a fruitful path for future research.

Another interesting avenue for future research is to examine the relationship of growth mindset and grit scores to self-talk frequency. Individuals who are high in grit scores should report more frequent self-talk than those who are low in grit, assuming that grit reflects a problem-solving orientation that helps to reduce cognitive disruption. Uhrich et al. (2023) examined undergraduate students' constructive or dysfunctional self-talk in response to hypothetical and real academic scenarios. They found that evidence that growth mindset and grit characterized constructive self-talk whereas fixed mindset and lack of grit characterized dysfunctional self-talk. However, this finding does not indicate whether self-talk is more likely with a growth mindset and higher grit scores and whether those with a fixed mindset and lower grit scores engage in less frequent self-talk.

Research finds that low self-concept clarity is associated with lower self-control scores (e.g., Jiang et al., 2023; Xiao et al., 2025). That finding suggests that self-control might play a role in helping people define their sense of self, with individuals who have a clear sense of self having higher self-control. Given the findings from Study 2 (higher self-control scores associated with more frequent self-talk), it may be that self-control plays a more complicated (e.g., indirect or mediating) role with respect to self-talk frequency. For example, if individuals use their self-control to help clarify their sense of self, they might end up not needing to talk to themselves about their self and identity as much.

The emphasis of cognitive-behavioral therapy (e.g., Dobson and Dozois, 2021) has historically been on the content of people's self-talk (e.g., its negative, irrational, or dysfunctional qualities). Given the evidence that cognitive disruptions are associated with increased self-talk, it seems promising that therapeutic interventions also consider individual differences in self-talk frequency. For example, individuals who frequently talk to themselves in response to specific situations might be more receptive to cognitive-behavioral therapy that requires the identification and recalibration of dysfunctional self-talk.

This set of studies has framed cognitive disruption as the primary mechanism linking traits to self-talk frequency. However, there are plausible alternative explanations for the relationships we found. For example, a variable such as negative affective state could account for significant amounts of the variance in our measures. At present, there is very little research on whether instances of emotional disruption are related to self-talk frequency. The automatic thoughts results from Study 3 suggest a possible role of emotion in self-talk content as well as frequency. It is conceivable that having a weak sense of self, lacking mindful awareness, having dissociative experiences, and having antisocial and psychopathy traits are emotionally as well as cognitively disruptive. Future research could explore the relative contributions of cognitive and emotional disruption to self-talk frequency.

In conclusion, the present set of studies provides good additional support for the prediction that tendencies toward cognitive disruption are associated with more frequent self-talk. Several possible avenues for future research can help to provide a more advanced and nuanced understanding of when and why people might talk more often to themselves under conditions of cognitive disruption.

## Data Availability

The raw data supporting the conclusions of this article will be made available by the authors, without undue reservation.
